# Complement activation in children with *Streptococcus pneumoniae* associated hemolytic uremic syndrome

**DOI:** 10.1007/s00467-021-04952-w

**Published:** 2021-02-04

**Authors:** Johannes Holle, Sandra Habbig, Alexander Gratopp, Anna Mauritsch, Dominik Müller, Julia Thumfart

**Affiliations:** 1 Department of Pediatric Gastroenterology, Nephrology and Metabolic Diseases, Charité - Universitätsmedizin Berlin, Augustenburger Platz 1, 13353 Berlin, Germany; 2 Department of Pediatrics, Faculty of Medicine and University Hospital Cologne, University of Cologne, Cologne, Germany; 3 Department of Pediatric Pulmonology, Immunology and Intensive Care Medicine, Charité – Universitätsmedizin Berlin, Berlin, Germany

**Keywords:** Hemolytic uremic syndrome, Complement, Eculizumab, Children, *Streptococcus pneumonia*

## Abstract

**Background:**

Hemolytic uremic syndrome caused by invasive pneumococcal disease (P-HUS) is rare in children and adolescents, but accompanied by high mortality in the acute phase and complicated by long-term renal sequelae. Abnormalities in the alternative complement pathway may additionally be contributing to the course of the disease but also to putative treatment options.

**Methods:**

Retrospective study to assess clinical course and laboratory data of the acute phase and outcome of children with P-HUS.

**Results:**

We report on seven children (median age 12 months, range 3–28 months) diagnosed with P-HUS. Primary organ manifestation was meningitis in four and pneumonia in three patients. All patients required dialysis which could be discontinued in five of them after a median of 25 days. In two patients, broad functional and genetic complement analysis was performed and revealed alternative pathway activation and risk haplotypes in both. Three patients were treated with the complement C5 inhibitor eculizumab. During a median follow-up time of 11.3 years, one patient died due to infectious complications after transplantation. Two patients showed no signs of renal sequelae.

**Conclusions:**

Although pathophysiology in P-HUS remains as yet incompletely understood, disordered complement regulation seems to provide a clue to additional insights for pathology, diagnosis, and even targeted treatment.

## Introduction

Hemolytic uremic syndrome (HUS) is the most common cause of acute intrarenal kidney injury in childhood, mainly caused by Shigatoxin-producing *Escherichia coli* (STEC-HUS) [1]. Non-STEC-HUS can be caused by anomalies in complement regulating genes or by acquired, secondary alternative complement pathway activation. One peculiar form of HUS is accompanied by invasive infections with *Streptococcus pneumoniae* (invasive pneumococcal disease, IPD). Although pneumococcal HUS (P-HUS) represents approximately 5–15% of all HUS cases [2], robust data about its clinical course, treatment, and outcome are lacking. New insights into disease pathophysiology of P-HUS suggesting a potential role of alternative complement pathway activation contributing to disease development have been reported [2], but clinical reports about complement activation in P-HUS are still rare.

Here, we provide a case series of P-HUS underlining the potential role of complement activation and, hereby, a potential treatment option.

## Patients and methods

We retrospectively analyzed clinical and laboratory data of the acute phase in children diagnosed with P-HUS between the years 1996 and 2019. Diagnosis of P-HUS, was based on recent recommendations including evidence for HUS (acute kidney injury, microangiopathic hemolytic anemia with schistocytes, thrombocytopenia), evidence of IPD (positive culture of blood or another sterile biological fluid), and absence of disseminated intravasal coagulation [2].

We investigated demographic (gender, age, body height and weight), microbiological (primary site of infection, results of bacterial cultures and serology), laboratory data (hemoglobin, platelet count, serum creatinine, Coombs test, complement diagnostics, when available, including genetics analyzing the following genes: *ADAMTS13*, *C3*, *CFB*, *CFH*, *CFHR-1-5*, *CFI*, *DGKE*, *MCP/CD46*, *MMACHC*, and *THMBD*) and clinical data. Further, the duration and modality of kidney replacement therapy was encountered as well as administration of the complement C5 inhibitor eculizumab. Follow-up parameters were analyzed including estimated GFR (eGFR) using the Schwartz formula [3], arterial hypertension (defined as blood pressure values above the 95th percentile in at least three individual measurements [4]), proteinuria (defined by the protein-creatinine ratio (g/g) in spot urine as absence of proteinuria (< 0.2 or negative) or positive proteinuria (> 0.2)), and the occurrence of relapses until the last documented visit.

## Results

Between 1996 and 2019, seven patients with definite diagnosis of P-HUS were treated at our hospital. Clinical presentation and outcome are listed in Table 1. Median patient’s age at presentation was 12 months [range 3–28 months].

**Table 1:** Clinical presentation, complement activation, therapy, and outcome of patients with P-HUS

Patient	No. 1	No. 2	No. 3	No. 4	No. 5	No. 6	No. 7
Initial presentation							
Age (months)	8	3	10	12	12	28	17
Gender	M	F	F	M	M	F	F
Weight (kg)	9.7	7.3	5.8	9.5	9.8	12.2	10.8
Height (cm)	75	66	67	78	76	90	82
Hb (g/dl)	8.6	6.5	8.9	5.1	9.6	10.9	10.7
Thrombocytes (/nl)	75	125	229	16	109	249	137
Creatinine (mg/dl)	2.4	2.5	1.2	3.1	2.3	1.0	2.2
Urea (mg/dl)	136	187	17	209	161	31	189
Fragmentocytes	+	+	+	+	+	+	+
Direct Coombs test	–	–	–	+	–	–	+
Primary manifestation of IPD	Meningitis	Meningitis	Meningitis	Pneumonia	Meningitis	Pneumonia	Pneumonia
Pneumococcal culture Pneumococcal serotype	CSF	Blood	Blood	Pleura	Blood/CSF S 15A	Blood S 3	Blood S 3
Complement diagnostics							
APH50 (60–140%) CH50 (74–151%) C3 (0.89–1.87 mg/ml) C3d (< 40 mU/l) C5b-9 (58–239 ng/ml) CFB (0.21–0.40 mg/ml) CFH (207–666 μg/ml) Genetics^a^	Not available	Not available	Not available	Not available	0.75	45 60 0.28 137 1160 0.6 227 MCP-H2^b^	67 n.d. 0.54 83 221 0.3 228 MCP-H2^b^
Therapy							
Dialysis modality Dialysis duration (days)	PD CKD 5	PD CKD 5	PD 20	PD 22	PD 42	PD + HD 31	PD 25
Eculizumab Eculizumab duration	–	–	–	–	Yes 1 dose	Yes 6 months	Yes 3 doses
Last follow-up							
Age Duration of follow-up eGFR (ml/min*1.73 m^2^) Arterial hypertension Proteinuria	18.0 years 17.3 years CKD 5 + +	3.8 years 2.6 years CKD 5^c^ + +	12.9 years 11.8 years 51 + +	12.3 years 11.3 years 93 – –	5.6 years 11.6 years 81 + –	5.5 years 3.2 years 111 – –	3.8 years 2.4 years 32 + +

*M* male, *F* female, *IPD* invasive pneumococcal disease, *CSF* cerebrospinal fluid, *MCP-H2* membrane cofactor-protein haplotype 2, *PD* peritonal dialysis, *HD* hemodialysis, *eGFR* estimated glomerular filtration rate, *CKD 5* stage 5 chronic kidney disease

^a^Genetics performed by means of next-generation sequencing analyzing *ADAMTS13, C3, CFB, CFH, CFHR-1-5, CFI, DGKE, MCP/CD46, MMACHC* and *THMBD*

^b^MCP-H2 GGAAC, heterozygote mutation

^c^Death

Acute phase: All patients showed acute kidney injury, hemolytic anemia with fragmentocytes, and thrombocytopenia within the acute phase of disease. Two patients had a positive Coombs test. IPD was present in all patients, with primary manifestation of meningitis in four patients and pneumonia in three patients. Despite vaccination with pneumococcal conjugate vaccine (PCV13), serotype S3 (which is included in PCV13) was detected in two patients, and serotype S15A was detected in one patient. Functional and genetic complement analyses were performed in two cases, revealing alternative pathway activation and a risk haplotype in the complement regulator membrane cofactor protein (*MCP-H2*, GGAAC, heterozygote mutation), but no other genetic anomalies in complement-regulating genes.

All patients needed kidney replacement therapy during the acute phase, which was performed as peritoneal dialysis. While five patients recovered during a median time on dialysis of 25 days [range 20–42], two patients developed stage 5 chronic kidney disease (CKD 5).

Three patients (patients 5, 6 and 7) were treated with the complement C5 inhibitor eculizumab due to initial signs of complement activation and/or supposed complement-mediated HUS. Patient no. 5 showed low serum C3 level. Further complement diagnostics were not performed. In this patient, eculizumab treatment was stopped after one dose due to severe infection and missing data about its effectivity in the year 2014. Patient no. 6 received eculizumab because of activated alternative complement pathway. Three days after first administration, thrombocyte count increased (from 32 to 45 thrombocytes/nl without transfusion) and LDH activity decreased (from 3373 to 1411 U/l) markedly. In the absence of known genetic anomalies in complement-regulating genes, eculizumab was discontinued 6 months after recovery. Patient no. 7 received eculizumab and showed rapid recovery similarly to patient no. 6 (thrombocytes from 66 to 183/nl and LDH activity from 4384 to 1908 U/l within two days). Eculizumab was discontinued after three doses as kidney function improved, and no genetic anomalies were detected.

Follow-up: During a median follow-up time of 11 years and 4 months (range 29 months–17 years, 4months), none of the patients experienced a relapse of P-HUS. Both patients with CKD 5 received a kidney transplant during the follow-up. One patient deceased due to infectious complications after transplantation. Three patients exhibited renal sequelae with reduced eGFR (*n* = 3), arterial hypertension (*n* = 3), and proteinuria (*n* = 2). Two patients recovered (eGFR > 90 ml/min/1.73 m^2^).

## Discussion

Within this study, we report about the clinical course and outcome of seven children with HUS, associated with IPD within a time period of 24 years. Despite national vaccination recommendations, IPD remains a major cause of morbidity and mortality in children living in Germany [5]. As reporting IPD is voluntary, exact data about the incidence of IPD are not available. Young children with a median age of 13 months are known to have the highest risk of IPD [5]. Hence, it is not surprising that the prevalence of P-HUS is highest in children under 2 years of age. Accordingly, the median age in our cohort was 12 months.

As reported previously, 92 patients with HUS were managed at our hospital between 1996 and 2004 [6], with evidence of P-HUS in five of them (5.4%). Therefore, the estimated rate of P-HUS recorded in our hospital (5.4%) is lower compared with the rate reported in the literature, implicating the need for resilient and reproducible diagnostic criteria [7, 8]. In contrast to other reports [9], we observed a higher proportion of meningitis compared with pneumonia. The finding of serotype S3 in two children despite having received appropriate vaccination (PCV13) underlines the potential risk of vaccination failure.

As discussed in a recent review article [2], the pathophysiology of P-HUS remains incompletely understood. The mechanism of endothelial damage by exposure of Thomsen-Friedenreich antigen, revealed by *S. pneumoniae* neuraminidase activity, remains widely accepted [2]. However, the subsequent binding of preformed IgM antibodies [10], resulting in a positive direct Coombs test [11], is more and more challenged by contradictory findings [2] and cannot be confirmed in our cohort with only two patients presenting with a positive Coombs test. Interestingly, alternative complement pathway activation might play a role in determining why some patients with IPD develop P-HUS while others do not. As a result of neuraminidase desialylation, factor H binding to C3 convertase decreases, resulting in alternative complement activation *in vitro* [12]. Moreover, there are reports on alternative complement pathway activation *in vivo* and partially pathogenic variants in complement regulating genes [13–15]. Our findings support an involvement of the alternative complement system in P-HUS pathology. Both patients who received comprehensive complement analysis showed significant activation of the alternative pathway during the acute phase. Genetic analysis revealed no definite pathogenic variants in complement-regulating genes. Nevertheless, the *MCP-H2* risk haplotype was found in both patients, reported to be associated with a 2–3-fold increased risk for developing aHUS and contributing to its pathophysiology in the context of the multiple-hits theory [16]. Three of our patients were treated with eculizumab. Eculizumab has been described in two patients with P-HUS previously [14, 17] — both showed rapid hematological recovery, and kidney replacement therapy could be discontinued.

P-HUS is associated with a high morbidity and mortality, indicated by 10.1% of patients developing CKD 5, 16% developing CKD and hypertension, and 12.3% deaths within the acute phase, especially in those cases with meningitis as primary manifestation [18]. In our cohort, no patient died during the acute disease course, and dialysis could be discontinued in five patients.

During the median follow-up time of 11 years and 4 months, two patients developed CKD 5 and three patients showed renal sequelae, while two patients showed recovery of kidney function. Of note, patient no. 6 who was treated for the longest period with eculizumab showed the most favorable outcome despite showing the most severe complement activation.

These findings indicate that besides other factors, the terminal pathway activation is critical for eculizumab to be effective in treatment of patients with P-HUS. Although complement activation data are lacking in five of seven patients, and accordingly, this limits the evidence of the study, our findings support the hypothesis of complement activation in P-HUS and favor the potential benefit of complement inhibition in selected patients.
